# In silico characterization of a novel putative aerotaxis chemosensory system in the myxobacterium, *Corallococcus coralloides*

**DOI:** 10.1186/s12864-018-5151-6

**Published:** 2018-10-19

**Authors:** Gaurav Sharma, Rebecca Parales, Mitchell Singer

**Affiliations:** Department of Microbiology and Molecular Genetics, University of California, Davis, California USA

**Keywords:** Chemotaxis, Energy taxis, MCP, Oxygen sensing, Aerotaxis, Signal transduction

## Abstract

**Background:**

An efficient signal transduction system allows a bacterium to sense environmental cues and then to respond positively or negatively to those signals; this process is referred to as taxis. In addition to external cues, the internal metabolic state of any bacterium plays a major role in determining its ability to reside and thrive in its current environment. Similar to external signaling molecules, cytoplasmic signals are also sensed by methyl-accepting chemotaxis proteins (MCPs) via diverse ligand binding domains. Myxobacteria are complex soil-dwelling social microbes that can perform a variety of physiologic and metabolic activities ranging from gliding motility, sporulation, biofilm formation, carotenoid and secondary metabolite biosynthesis, predation, and slime secretion. To live such complex lifestyles, they have evolved efficient signal transduction systems with numerous one- and two-component regulatory system along with a large array of chemosensory systems to perceive and integrate both external and internal cues.

**Results:**

Here we report the in silico characterization of a putative energy taxis cluster, Cc-5, which is present in only one amongst 34 known and sequenced myxobacterial genomes, *Corallococcus coralloides.* In addition, we propose that this energy taxis cluster is involved in oxygen sensing, suggesting that *C. coralloides* can sense (either directly or indirectly) and then respond to changing concentrations of molecular oxygen.

**Conclusions:**

This hypothesis is based on the presence of a unique MCP encoded in this gene cluster that contains two different oxygen-binding sensor domains, PAS and globin. In addition, the two monooxygenases encoded in this cluster may contribute to aerobic respiration via ubiquinone biosynthesis, which is part of the cytochrome bc1 complex. Finally, we suggest that this cluster was acquired from Actinobacteria, Gammaproteobacteria or Cyanobacteria. Overall, this in silico study has identified a potentially innovative and evolved mechanism of energy taxis in only one of the myxobacteria, *C. coralloides*.

**Electronic supplementary material:**

The online version of this article (10.1186/s12864-018-5151-6) contains supplementary material, which is available to authorized users.

## Background

Bacteria actively sense their rapidly changing environment and alter their behavior in response. One important environmental challenge in soil is the ever-changing oxygen level. Depending upon the nature and behavior of the bacterium, cells will either move away or towards increasing concentrations of oxygen [1]. Bacteria can perceive and respond to environmental signals using a vast array of diverse chemoreceptor proteins and transmit the signals to other cytoplasmic signaling proteins present downstream in the signaling pathway. These clusters of proteins that receive, transmit and respond to the signal constitute a chemosensory system (CSS), which primarily controls chemotaxis i.e. directed motility in response to a chemical gradient [2–6]. Chemotaxis is predominantly conserved throughout the bacterial and archaeal kingdoms. These multiprotein systems receive the sensory environmental signal using methyl-accepting chemotaxis proteins (MCPs) [7] as the receiver and initial transmitter of the chemotactic signal. The MCP transmits the signal to CheW, which in turn transfers the signal from the MCP to CheA, a histidine kinase that undergoes auto-phosphorylation. CheA~P in turn phosphorylates CheY. CheY~P interacts with the motility system and regulates the motility apparatus via flagella or pili [8–10]. Reversible methylation and demethylation of MCPs via CheR and CheB, respectively, play an important role in chemotaxis adaptation or memory, by adjusting the MCP’s sensitivity for new signals [for reviews see [2, 6, 7, 11, 12]].

With the boom in next generation sequencing and genomic studies, the scientific literature is flourishing with the identification of diverse types of chemosensory system organizations whose functions are not only limited to chemotaxis, but also range to diverse alternative cellular functions [6, 13] such as sporulation [14], biofilm formation [15, 16], exopolysaccharide (EPS) production [17–19], and flagellum biosynthesis [20], for example. For each function, the bacteria need a different type of intracellular or extracellular signaling mechanism and a dedicated MCP. For example, the MCP protein WspA in *Pseudomonas aeruginosa* has a 4HB domain (ligand binding domain) between two predicted transmembrane helices, and is used to sense the signal for biofilm formation and responds by activating c-di-GMP production [15]. Similarly, Mcp3A and Mcp3B in *Myxococcus xanthus*, which have HAMP and MCPsignal domains with three predicted transmembrane helices and no ligand binding domain, sense a yet unknown signal(s) for sporulation, causing early aggregation amongst the starving cells [21].

Intracellular behavior inside any bacterial cell is highly dynamic and regulated via complex protein-protein and protein-DNA interactions. Amongst all types of intracellular and extracellular signals, the physiological state or the internal energetic condition is one of the major determinants of a suitable niche for any bacterium. Owing to this, a motile bacterial cell will navigate from a niche in which it displays low metabolic activity (less favorable) to one that supports higher metabolic activity (more favorable) [22]. This concept has been termed energy taxis [22–25]. Several compounds, including the substrates or products of diverse metabolic pathways, such as sugars, amino acids, oxygen, nitrate, etc., have been suggested to function as energy taxis signal molecules. [25]. Considering the different behaviors, habitats and metabolic requirements, diverse mechanisms of energy taxis have been reported including aerotaxis, phototaxis, redox taxis, and taxis towards electron acceptors [23, 25]. Theoretically, energy taxis might be present in all motile microbes, although it has only been reported in a few species and demonstrated as a dominant behavior in even fewer; one example is *Azospirillum brasilense* [23]. The primary proteins involved in energy taxis are MCPs and MCP-like proteins, which have diverse ligand binding domains (LBD) that differ for diverse types of energy taxis. Considering the latest classification based on the distribution of LBD and transmembrane (TM) domains, MCP proteins can be divided into seven topologies (Ia, Ib, II, IIIm, IIIc, IVa, and IVb) [26, 27]. MCP proteins with TM domains generally sense extracellular signals, whereas those lacking TM domains (cytoplasmic MCPs) sense intracellular signals such as those involved in energy taxis [12, 25, 26]. Diverse LBD domains involved in various energy taxis functions include PAS domains for sensing oxygen, redox potential, small ligands, and cumulatively energy levels of a cell [28–30], GAF domains for sensing light [31], and globin domains for direct oxygen sensing [32]. Among these, aerotaxis, whereby the cells move towards or away from oxygen in search of an optimal oxygen concentration for their metabolic state, is the most studied form of energy taxis. Aerotaxis has primarily been explored in *Escherichia coli*, *Pseudomonas aeruginosa* and *Bacillus subtilis*, which require the Aer, TlpC/TlpG and HemAT MCP proteins, respectively, as oxygen sensors. Aer and TlpC/TlpG MCP receptors contain a PAS domain (an acronym for Drosophila period clock protein [**P**ER], vertebrate aryl hydrocarbon receptor nuclear translocator [**A**RNT], and Drosophila single-minded protein [**S**IM]) motif) [33], which have a prosthetic group binding pocket to bind molecular oxygen and use flavin adenine dinucleotide (FAD) as a cofactor. FAD aids in oxygen binding and redox sensing [30, 34, 35]. The HemAT MCP receptor has a Protoglobin (PF11563) domain, a member of the globin superfamily (other members include: Globin (PF00042), Bac_globin (PF01152) etc.), which acts as an oxygen sensor [36].

Myxobacteria are well known for their large genome size (9–16 Mbps), high GC content (~ 70%), and their complex and social behavioral phenotypes; including gliding motility, sporulation, biofilm formation, predation, secondary metabolite production, and large biomolecule degradation [37–43]. All these unique physiological and metabolic features, accommodated in a single cell, make them one of the most complex groups of bacteria in the bacterial domain [44]. They are also well known for encoding the largest number of one- and two-components signal transduction systems and chemosensory systems (CSS, up to 12) [13, 37, 45–47]. Considering the genomic and metabolic complexity of the myxobacteria, we are continuously mining these genomes for information regarding potential functions for these different CSS. Here we have identified a potentially novel energy taxis gene cluster in one of the myxobacteria, *Corallococcus coralloides* DSM 2259. *C. coralloides* encodes 12 chemosensory systems*,* among which one system has a unique set of proteins that is predicted to be involved in oxygen sensing. Amongst 34 sequenced myxobacterial genomes, *C. coralloides* is the only species that encodes such a CSS cluster. Our study suggests that *C. coralloides* has procured this complete energy taxis cluster from Gammaproteobacteria and Actinobacteria via horizontal gene transfer.

## Methods

Order Myxococcales genomes [13, 37, 48–67] were downloaded from NCBI followed by gene prediction and functional annotation with RAST [68]. To identify functional domains, all proteomes were scanned against the Pfam-A v29.0 database [69] (downloaded on Oct 26, 2016) using hmmscan (maximum E-value 1e^− 5^) from the HMMER suite (http://hmmer.org/) [70] and further parsed using hmmscan-parser.sh. For phylogenetic analysis, all protein sequences were subjected to Basic Local Alignment Search Tool (BLASTp) [71] against the non-redundant protein (NR) database (downloaded on 10-21-2016) with defined cutoff values: [maximum 1e^− 5^ E-value, minimum 35% query coverage and minimum 35% similarity]. The protein sequences of top 100 homologs were extracted and aligned using MUSCLE v3.8.31 [72]. The alignment was used to generate a maximum likelihood phylogeny using RAxML version 8.2.4 [73] using following parameters: Jones–Taylor–Thornton (JTT) protein substitution model, Gamma Distributed Rates among sites, Randomized Maximum Parsimony (MP) method for tree optimization, and 100 times bootstrapping. The obtained best maximum likelihood (ML) tree was visualized in iTOL [74] and the domain and taxonomy distribution was mapped onto the tree. Based on the location of genes/domains within a respective chromosome/contig/protein, the module organization of a cluster and the Pfam domain organization of a protein were identified followed by the making of maps using IBS 1.03 software [75]. We have also downloaded the dataset of 8,075 complete bacterial genomes from NCBI reference as on Nov 6, 2017. Subsequently using the above strategy, we have identified the Pfam domain organization of all 8,075 proteomes.

## Results

### Distribution of ‘PAS’ and ‘MCPsignal’ domains

PAS domains are well known to sense oxygen, redox potential and light, and they have been shown to be involved in taxis behavior, development, circadian rhythmicity, and regulation of metabolism [11, 30, 76, 77]. Similarly, ‘MCPsignal’ [Pfam: PF00015] domains function as chemoreceptors for diverse signals [11, 26]. Before identifying their distribution in our model organisms i.e. myxobacteria, we want to understand how these domains are distributed across Eubacterial kingdom. Therefore we scanned 8,075 complete genomes downloaded from NCBI and identified PAS domains in almost 90% (7,226) of the genomes; many are associated with regulatory domains such as HisKA, HATPase_c, Response_reg, HTH, MCPsignal, etc. We found that *Desulfovibrio* (141), *Archangium* (141), *Microcoleus* (132), *Sorangium* (120), *Desulfatibacillum* (118), *Desulfomonile* (113), *Methylobacterium* (108), *Oscillatoria* (105), *Cystobacter* (104), and *Magnetospirillum* (98) have the largest number of proteins with one or more PAS domains per genome. Similarly, we identified the number of proteins having ‘MCPsignal’ domains per genome and found that they are present in ~ 4,639 genomes (~ 60%) with maximum representation (> 60) in *Azospirillum* (89), *Clostridium* (86), *Aquaspirillum* (73), *Herbaspirillum* (71), *Pararhodospirillum* (67), *Magnetospirillum* (62), *Methylobacterium* (61), *Pseudomonas* (60), *Bradyrhizobium* (60), *Aliivibrio* (59), and *Desulfovibrio* (58). The MCPsignal domain is most frequently associated with HAMP domains (which assist in transferring the signal to the former), and several other ligand binding Pfam domains such as 4HB_MCP_1, dCache_1, TarH, PAS_3, sCache_2, PAS_9, CZB, HBM, Protoglobin, PAS_4, Cache_3-Cache_2, PilJ, CHASE3, etc., supporting the previous findings [12]. The functional role of MCP proteins is determined based on the ligand binding and the presence or absence of transmembrane domains. PAS domains have been reported to have important roles in energy taxis via responding to oxygen, light and voltage [28, 30, 34, 76]. We found that 3,165 (~ 40%) out of 8,075 genomes encode PAS and MCPsignal domains together within the same protein, with maximum numbers in *Desulfovibrio* (17), *Aquaspirillum* (12), *Methylobacterium* (10), *Pseudomonas* (9), *Pseudodesulfovibrio* (9), *Marinomonas* (8), *Halomonas* (8), *Gemmata* (8), *Alteromonas* (8), and *Vibrio* (7). We believe that these proteins are involved in various energy taxis related functions regulating growth of the bacterium according to their energy state in their respective niche.

Myxobacteria are a complex group of bacteria that share a variety of unique phenotypic features such as large genome size [37, 48, 54, 58], production of secondary metabolites [43, 78–81], and their developmental phenotype [44, 62, 82–85]. Considering their genomic and physiological features, we identified the distribution of PAS and MCPsignal domains amongst them in search of any putative energy taxis mechanisms. We found that all myxobacterial organisms have from 13 to 145 proteins with single or multiple PAS domains (Table 1). Most of the myxobacteria are strict aerobes and have genomes > 9 Mb in size. The exceptions include *Pajaroellobacter*, *Anaeromyxobacter* and *Vulgatibacter,* which have small genomes, between 2 and 6 Mbp [66, 86, 87]. Among these, *Pajaroellobacter*, a strict anaerobic pathogen of cows, have no proteins with a PAS domain. *Anaeromyxobacter* and *Vulgatibacter* both have small myxobacterial genomes but the former is a facultative anaerobe [86, 88] whereas the latter is a strict aerobe [87]. We found significant variations in PAS containing proteins amongst the three-myxobacterial suborders. We also found that marine myxobacteria i.e. *Enhygromyxa*, *Haliangium*, and *Plesiocystis* have fewer PAS containing proteins as compared to the terrestrial myxobacteria. Our study also suggested that there is no correlation between genome size and the number of PAS domain containing proteins. Similarly, we examined the distribution of MCPsignal proteins and found that members of the suborder Cystobacterineae in the order Myxococcales have ~ 20–32 MCP proteins per organism (with the exception of *Anaeromyxobacter* and *Vulgatibacter*), whereas the members of suborder Nannocystineae and Sorangiineae, which have 10–16 Mb genomes, have only 2–5 MCP proteins per organism (Table 1). This is in accordance with our study of chemosensory systems in myxobacteria [13]. The strict anaerobic pathogen *Pajaroellobacter* does not encode any MCP proteins. Interestingly, none of the MCP proteins encoded by any of the myxobacteria has a PAS domain except for *Corallococcus coralloides*. This unique myxobacterial MCP protein in *C. coralloides* (Cc_4972; WP_014397685.1) is a part of organized CSS cluster with all constituent proteins whose architecture was also shown in our previous study [13]. This study will further highlight the putative role of this CSS in energy taxis and its putative evolution within a single myxobacterial species, *C. coralloides*.

**Table 1:**
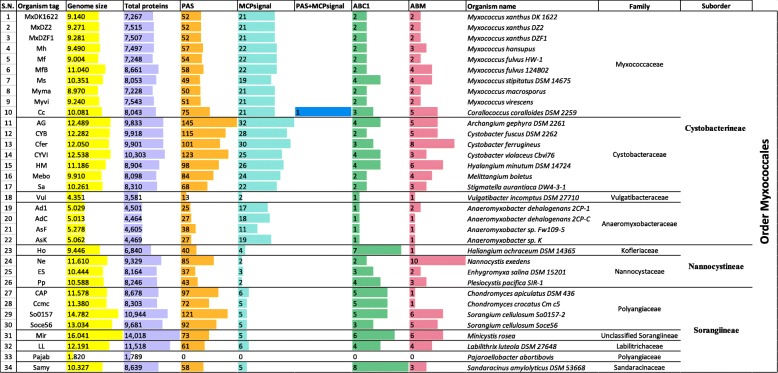
Distribution of proteins with ‘PAS’, ‘MCPsignal’, ABC1 and ABM domains amongst order Myxococcales genomes

### Identification of a cytoplasmic energy taxis chemoreceptor in *C. coralloides*

**Fig. 1:**
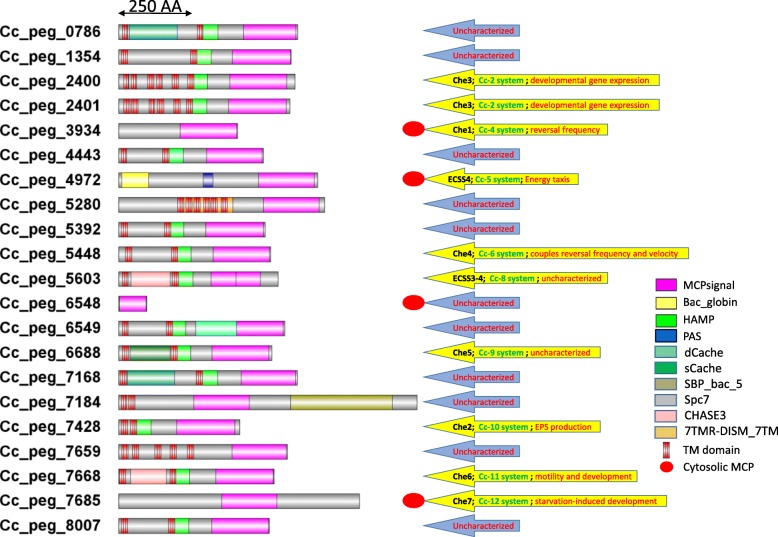
Domain architecture of twenty-one chemoreceptors in *C. coralloides*. Pfam domains (names in the bottom-right corner) are mapped throughout the protein sequence with scale as given above. Arrows in the right side indicate categorization of the CSS to which the respective MCP protein belongs, along with its putative functional roles

Myxobacterial genomes are well known to encode large numbers of chemosensory systems, which are involved in regulating diverse physiologic functions [6, 13, 47]. We previously reported that the *C. coralloides* genome encodes 12 CSS and 21 MCP proteins [13]. Among the 12 CSS, three CSS (Che8, ACSS2 and ACSS3) do not have an associated MCP protein and Che3 has two MCP proteins in a single system. We found that ten amongst 21 MCP proteins in *C. coralloides* are constituents of a CSS, presumably sensing and responding back using their own chemosensory system; whereas the other eleven MCP genes are scattered in the genome **(**Fig. 1**)**. It has been suggested that completely organized CSS systems can also perceive signals received by scattered MCP proteins similar to the signals from the MCPs encoded together [47, 89]. *C. coralloides* has not been used as a model organism to study chemosensory systems. Based on the research performed with the model organism *Myxococcus xanthus*, six out of the 12 *C. coralloides* CSS have been assigned a functional role according to homology studies [13] and the rest of them are uncharacterized **(**Fig. 1**)**.

**Fig. 2:**
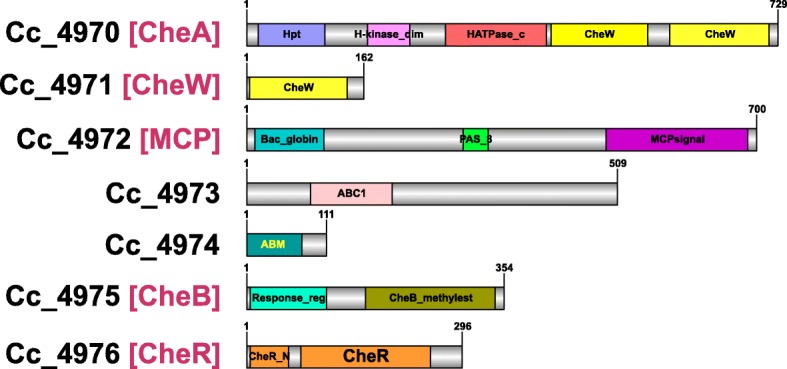
The modular organization of energy taxis cluster in *C. coralloides*. Pfam domains are mapped on the protein sequence with scale. Corresponding protein ids for all proteins are Cc_4970 (WP_014397683.1), Cc_4971 (WP_014397684.1), Cc_4972 (WP_014397685.1), Cc_4973 (WP_014397686.1), Cc_4974 (WP_014397687.1), Cc_4975 (WP_014397688.1), and Cc_4976 (WP_014397689.1)

The presence of a PAS domain along with a MCPsignal domain makes the *C. coralloides* MCP protein (Cc_4972) unique amongst the myxobacteria. The Pfam domain analysis of Cc_4972 revealed an additional interesting characteristic of this protein: the presence of a Bac_globin domain (PF01152), which is a characteristic of heme-containing globular proteins involved in oxygen binding/transport **(**Figs. 1 and 2**)**. The presence of these three domains in a single protein represents a rare combination that was identified only in seven such proteins [in *Corallococcus coralloides* DSM 2259 (Deltaproteobacteria), *Colwellia sp.* MT41, *Glaciecola nitratireducens* FR1064, *Halioglobus japonicus* (Gammaproteobacteria), *Nitrospira defluvii* (Nitrospirae), *Phenylobacterium zucineum* HLK1 (Alphaproteobacteria), *Rubinisphaera brasiliensis* DSM 5305 (Planctomycetes)] amongst the 8,075 complete genomes. It is postulated that the PAS domain of the *E. coli* Aer receptor uses FAD to monitor/sense altered redox conditions in the cytoplasm, whereas *B. subtilis* performs aerotaxis by sensing oxygen directly via the HemAT protein protoglobulin domain, which contains a bound heme [12, 29, 32, 90]. It has also been suggested that the Aer chemoreceptor directly binds a heme moiety as a cofactor to bind oxygen [35]. Considering these arguments, the combination of PAS, Bac_globin, and MCPsignal domains in the *C. coralloides* MCP protein marks it a special aerotaxis sensor (according to present literature). Cc_4972 is a cytosolic protein with no transmembrane domains (as identified using TMHMM Server v. 2.0 program) **(**Fig. 1**)** and it should be classified in the MCP-IVa category based on the previous report [26].

### Identification of an energy taxis chemosensory system in *C. coralloides*

Interestingly, Cc_4972 is present in a well-organized chemosensory system gene cluster (Cc-5 CSS; WP_014397683.1-WP_014397689.1). In our previous study [13], it was classified as Extra CSS-4 (ECSS4), which has a different modular architecture as compared to other myxobacterial CSS and separate phylogenetic positioning in the CheA and CheB phylogenetic trees. Cc-5 has seven constituent proteins (CheA-CheW-MCP-x-x-CheB-CheR; WP_014397683.1-WP_014397689.1) among which five are chemosensory proteins and two are hypothetical proteins **(**Fig. 2**)**. We could not identify a CheY response regulator protein encoded nearby. Interestingly, Pfam domain analysis suggested the presence of ABC1 and ABM domains, respectively, in the two hypothetical proteins. Both of these proteins are distantly related to sensing oxygen or have functions related to aerobic respiration [91, 92].

ABC1 proteins belong to the Eukaryotes-like protein kinase superfamily, which are widespread in myxobacteria [93, 94]. Their role in cellular regulation and signal transduction is well documented in the bacterial kingdom especially in myxobacteria [95, 96]. These ABC1 proteins include AarF from *Providencia stuarti* and YigR from *E. coli*, which have been proposed to function as ubiquinone (cofactor Q) biosynthesis monooxygenases [91, 97]. The ABC1 protein in *Saccharomyces cerevisiae* (*ScCOQ8*) was found to be essential for redox activity, and mutations in this gene resulted in defects in aerobic respiration due to the absence of quinones, and further leading to the instability of the cytochrome bc1 complex [98]. The ABC1 protein in *Arabidopsis* chloroplasts was reported to play important roles in oxidative stress balance/tolerance [99]. These family proteins in yeast are also predicted to be novel chaperonins and known to work as a suppressor of cytochrome b mRNA translation defect [98]. We found that one to eight ABC1 protein homologs are encoded per myxobacterial genome. A single homolog is present in *Anaeromyxobacter* and *Vulgatibacter,* whereas in other myxobacteria multiple homologs are present with no apparent synteny.

Antibiotic biosynthesis monooxygenase (ABM) domain containing proteins have been reported to be involved in metabolism, translation/transcriptional control and antibiotic biosynthesis [92]. This domain was first identified in the *Streptomyces coelicolor* monooxygenase, ActVA-Orf6, which oxidizes phenol groups to quinones and therefore participates in antibiotic actinorhodin biosynthesis [92]. It has also been suggested that ABM proteins help in maintaining the equilibrium of quinones required for the electron transport chain and simultaneously reduce the toxic free radicals production by quinones and quinols [100]. These proteins have a ferredoxin-like fold and can carry out oxygenation in the absence of any prosthetic groups, metal ions or cofactors, which are generally associated with activation of molecular oxygen. Besides this, a few monooxygenases in *M. tuberculosis* (Rv0793) work as reactive oxygen species scavengers, which is beneficial in evading host defenses [101]. Besides these, ABM family proteins are also known to function as heme-degrading enzymes in *Staphylococcus aureus* and signal transduction protein in *Staphylococci*. We found that similar to ABC1 proteins, ABM domains are encoded in almost all myxobacterial genomes, ranging from one to ten homologs per genome. To our surprise, the maximum representation was found in a soil myxobacterium, *Nannocystis exedens*, from the suborder Nannocystineae that has mostly marine organisms. With the exception of *Anaeromyxobacter*, *Vulgatibacter*, *Haliangium*, and *Chondromyces*, all have multiple ABM proteins.

### Evolution of *C. coralloides* aerotaxis chemosensory system

**Fig. 3:**
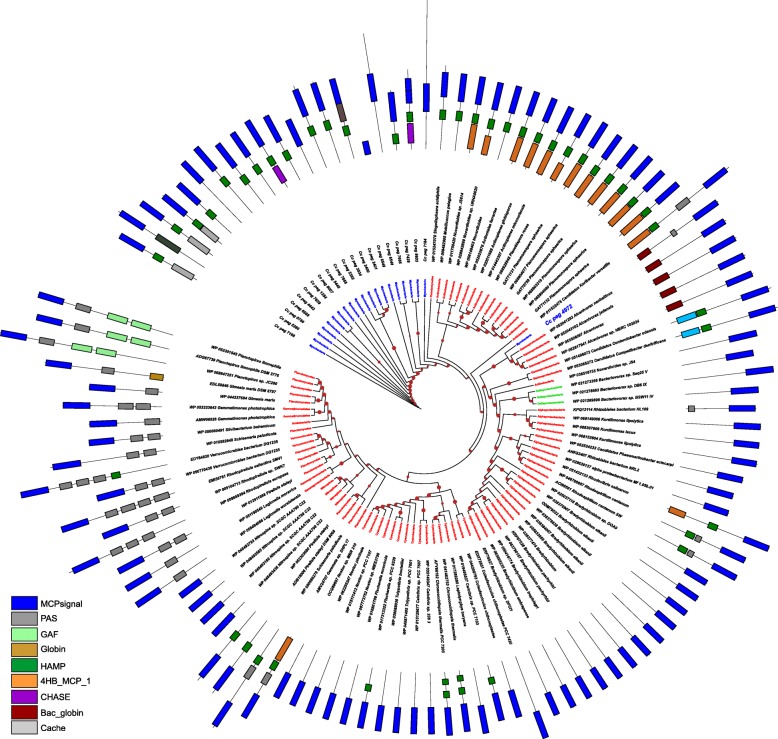
Maximum likelihood phylogeny for *C. coralloides* MCP proteins. The top homologs of the MCP protein involved in energy taxis and rest of the *C. coralloides* MCPs were used to generate this represented Maximum likelihood (ML) phylogenetic tree with organism names in the center ring. The taxonomy lineage at the phylum level is represented in the innermost ring where myxobacterial homologs are in blue text, non-Myxococcales Deltaproteobacteria in green text and other taxa are in red text. The outermost ring presents the Pfam domain organization of each MCP according to their sequence length, and domain color codes are shown in the lower-left corner. Bootstrap values are provided corresponding to the tree nodes as red circles with sizes ranging from one (BS value 1) to 15 (BS value 100)

On closer analysis of the Cc-5 CSS cluster, we determined that all seven proteins encoded in the cluster (CheA-CheW-MCP-ABC1-ABM-CheB-CheR) are unique to *C. coralloides* and show no close sequence identity in any of the myxobacteria or even in the Deltaproteobacteria. We found that synteny was lost in the members of family Myxococcaceae and Cystobacteraceae, who are close relatives of *C. coralloides,* although it was conserved in upstream and downstream regions of this cluster. Therefore, we decided to examine the relatedness of the Cc5 CSS cluster proteins in other bacterial lineages using maximum likelihood phylogeny. Based on top BLASTp hits against the NR database, we generated phylogenetic trees with 100 bootstrap values. The CheA protein (Cc_4970) phylogeny indicated that branches with lineages from Alphaproteobacteria and Gammaproteobacteria share a common ancestor with the *C. coralloides* CheA protein (Additional file 1: Figure S1). A phylogenetic tree based on the CheW protein (Cc_4971) suggested that Actinobacteria, Planctomycetes and Alphaproteobacteria have a common relative with the Cc-5 CheW homolog from *C. coralloides* (Additional file 2: Figure S2). For the MCP protein phylogeny, we used the Cc-5 MCP (Cc_4972) and its top non-redundant (NR) dataset hits along with other *C. coralloides* MCPs as outgroups **(**Fig. 3**)**. MCP phylogeny clearly demarked the Cc-5 MCP from the rest of the MCPs, which were present together near the root. The Cc-5 MCP shared sister clades with Gammaproteobacteria homologs and share common ancestors with Acidobacteria and Actinobacteria homologs. Similar to the Cc-5 MCP Pfam organization, their sister terminal node Gammaproteobacteria members also have Bac_globin domains, while a PAS domain was not identified in the latter. The phylogeny of the ABC1 protein from the Cc-5 cluster suggested that the closest homologs were in the Gammaproteobacteria, Nitrospinae, Planctomycetes, and Actinobacteria (Additional file 3: Figure S3). BLAST homology searches of the ABM protein (Cc_4974) revealed that most of its closest homologs have an archaeal lineage rather than a Eubacterial lineage. Phylogeny also revealed Planctomycetes and Nitrospinae to be sister branches of the *C. coralloides* ABM protein, whereas archaeal homologs share common ancestral relatives with them (Additional file 4: Figure S4). The Cc-5 CheB (Cc_4975) protein phylogeny depicted Gammaproteobacteria as a sister clade, suggesting the latter to be closest relatives of *C. coralloides* (Additional file 5: Figure S5). CheR (Cc_4976) phylogeny showed that homologs from *C. coralloides* and Alphaproteobacteria share common ancestors with those from Actinobacteria (Additional file 6: Figure S6).

**Fig. 4:**
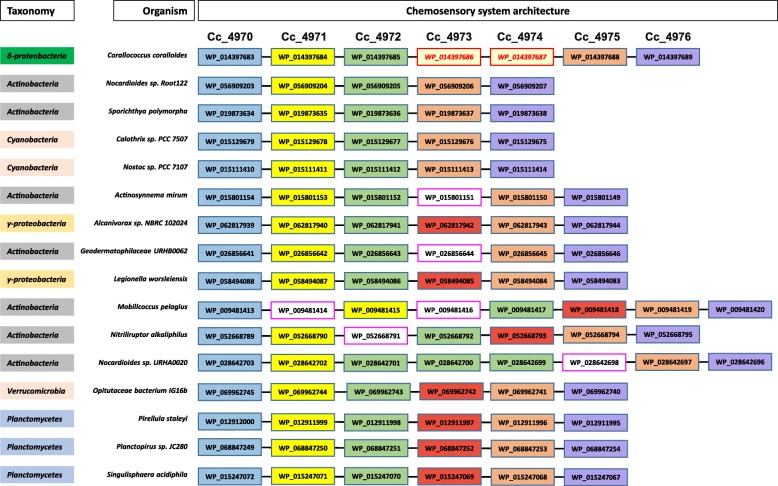
Horizontal gene transfer based procurement of complete energy taxis cluster in *C. coralloides:* Based on homology studies, homologs of all Cc-5 cluster proteins were extracted for each organism and arranged according to their chromosome location. With the exception of Cc_4973 and Cc_4974, the other proteins were predicted to have evolved in *C. coralloides* as a group following horizontal gene transfer

Based on BLASTp homology searches above the cutoff values, we identified the chromosomal location of all homologs of the Cc-5 cluster proteins and mapped those proteins with each other to find the putative clusters in respective organisms **(**Fig. 4**)**. Remarkably, we identified several clusters in different lineages i.e. Actinobacteria, Cyanobacteria, Gammaproteobacteria, Planctomycetes etc., where most of the genes shared similar cluster organizations to the Cc-5 cluster. However, we did not find any genes encoding homologs of the two non-CSS proteins i.e. ABC1 and ABM proteins as a part of any of the putative clusters. The most closely related clusters in *Nocardioides* sp. Root122, *Sporichthya polymorpha*, *Calothrix* sp. PCC 7507, and *Nostoc* sp. PCC 7107 have all five CSS homologs in a well-organized cluster. Based on our results, we suggest that these clusters were horizontally transferred to *C. coralloides*, and later on the non-CSS, i.e. ABC1 and ABM genes were procured from other bacterial groups, and became functional members of this cluster.

## Discussion

The present study provides strong suggestive evidence for energy taxis in this complex bacterium. The presence of PAS, Bac_globin and MCPsignal domains together suggest that the Cc-5 MCP protein (Cc_4972) is a novel cytoplasmic aerotaxis receptor. Based on these Pfam functional domains, we predict that this CSS can sense oxygen more efficiently than systems with only PAS-based or globin-based MCP proteins. Further, experimental studies will be required to confirm the function of this chemosensory system and to examine into the roles of proteins with ABC1 and ABM Pfam domains, which we believe play an important role in aerotaxis in *C. coralloides*. Our domain distribution studies demonstrate that myxobacterial MCPSignal proteins do not consist of any PAS domains; this leads to two contrasting hypotheses. The first is that most of the myxobacteria lost this MCP protein and corresponding CSS cluster, while *Corallococcus* still maintains it in a rudimentary or non-functional form, potentially on its way to loss. Alternatively, the other hypothesis is that the aerobic myxobacteria have unknown intrinsic mechanisms to pursue energy taxis, similar to the one as identified here in *C. corraloides*, that is acquired from other bacterial groups. We believe that with the identification of novel energy taxis domains/mechanisms, genome sequencing of new myxobacterial strains, and experimental studies, the intrinsic energy taxis mechanisms would be identified amongst these bacteria in the future. Overall, this study highlights the identification of a novel energy taxis MCP protein and its associated chemosensory system, which might be an efficient way for aerotaxis owing to the presence of PAS and Globin domains together.

## Conclusion

Sensing and responding back to environmental signals has been studied thoroughly among the bacterial kingdom, whereas sensing the internal ambiance is still limited to a few organisms. Here, we performed a computational characterization of a novel energy taxis cluster (Cc-5 CSS) in one of the myxobacteria, *C. coralloides*. Although, myxobacteria are known to be highly complex owing to their numerous physiological and metabolic activities, we could identify only one energy taxis cluster amongst 34 studied myxobacterial genomes most of which have > 9 Mb genomes and > 7000 proteins each. We report that this cluster has a MCP protein with both PAS and Bac_globin domains, which is a novel combination in itself and may have an advantage in oxygen sensing as compared to those with single PAS or single globin domains. We also identified the presence of two proteins with ABC1 and ABM domains, respectively, which are predicted to assist in ubiquinone biosynthesis and aerobic respiration via the cytochrome bc1 complex. We also suggest that this cluster of genes may have been acquired from Actinobacteria, Gammaproteobacteria or Cyanobacteria. The presence of this novel aerotaxis cluster [especially the single MCP sensor protein with PAS and globin domains] in one of the myxobacteria raises two important open questions for the scientific community; first, how oxygen sensing evolved amongst the myxobacteria as compared to their Deltaproteobacterial lineage and second, how other myxobacteria recognize and efficiently respond to the availability of oxygen.

## Additional files

Additional file 1: **Figure S1.** Maximum likelihood phylogeny for CheA protein of the energy taxis cluster in *C. coralloides*. The top homologs of the CheA protein (Cc_4970) involved in energy taxis in *C. coralloides* are used here to generate this represented ML phylogenetic tree with organism names in the outermost ring. The taxonomy lineage at the phylum level is represented in the inner ring where myxobacterial homologs are in blue text, non-Myxococcales Deltaproteobacteria in green text and other taxa in red text. Bootstrap values are provided corresponding to the tree nodes as blue circles with sizes ranging from one (BS value 1) to 15 (BS value 100). (PDF 49 kb)

Additional file 2: **Figure S2.** Maximum likelihood phylogeny for CheW protein, a part of the energy taxis cluster in *C. coralloides*. The top homologs of the CheW protein (Cc_4971) involved in energy taxis in *C. coralloides* were used to generate this represented ML phylogenetic tree with organism names in the outermost ring. The taxonomy lineage at the phylum level is represented in the inner ring where myxobacterial homologs are in blue text, non-Myxococcales Deltaproteobacteria in green text and other taxa in red text. Bootstrap values are provided corresponding to the tree nodes as blue circles with sizes ranging from one (BS value 1) to 15 (BS value 100). (PDF 51 kb)

Additional file 3: **Figure S3.** Maximum likelihood phylogeny for the Cc_4973 protein, a constituent of the Cc-5 energy taxis cluster. The top homologs of the Cc_4973 protein, which is encoded in the energy taxis cluster in *C. coralloides,* were used to generate this represented ML phylogenetic tree with organism names in the outermost ring. The taxonomy lineage at the phylum level is represented in the inner ring where myxobacterial homologs are in blue text, non-Myxococcales Deltaproteobacteria in green text and other taxa in red text. Bootstrap values are provided corresponding to the tree nodes as blue circles with sizes ranging from one (BS value 1) to 15 (BS value 100). (PDF 43 kb)

Additional file 4: **Figure S4.** Maximum likelihood phylogeny for the Cc_4974 protein, a constituent of the Cc-5 energy taxis cluster. The top homologs of the Cc_4974 protein, which is encoded in the energy taxis cluster in *C. coralloides,* were used to generate this represented ML phylogenetic tree with organism names in the outermost ring. The taxonomy lineage at the phylum level is represented in the inner ring where myxobacterial homologs are in blue text, non-Myxococcales Deltaproteobacteria in green text and other taxa in red text. Bootstrap values are provided corresponding to the tree nodes as blue circles with sizes ranging from one (BS value 1) to 15 (BS value 100). (PDF 36 kb)

Additional file 5: **Figure S5.** Maximum likelihood phylogeny for the CheB protein involved in the energy taxis cluster in *C. coralloides*. The top homologs of the CheB protein (Cc_4975) involved in the energy taxis in *C. coralloides* were used to generate this represented ML phylogenetic tree with organism names in the outermost ring. The taxonomy lineage at the phylum level is represented in the inner ring where myxobacterial homologs are in blue text, non-Myxococcales Deltaproteobacteria in green text and other taxa in red text. Bootstrap values are provided corresponding to the tree nodes as blue circles with sizes ranging from one (BS value 1) to 15 (BS value 100). (PDF 52 kb)

Additional file 6: **Figure S6.** Maximum likelihood phylogenetic tree for the CheR protein in the Cc-5 cluster in *C. coralloides*. The top homologs of the CheR protein (Cc_4976) involved in energy taxis in *C. coralloides* were used to generate this represented ML phylogenetic tree with organism names in the outermost ring. The taxonomy lineage at phylum level is represented in the inner ring where myxobacterial homologs are in blue text, non-Myxococcales Deltaproteobacteria in green text and other taxa in red text. Bootstrap values are provided corresponding to the tree nodes as blue circles with sizes ranging from one (BS value 1) to 15 (BS value 100). (PDF 50 kb)

Additional file 7: Cc-5 Locus Information File. (XLS 37 kb)

Additional file 8: File with NCBI web links from where Genome data could be downloaded. (XLS 44 kb)

## Abbreviations

BLAST: Basic Local Alignment Search Tool
CSS: Chemosensory System
MCP: Methyl-accepting chemotaxis protein
NCBI: National Center for Biotechnology Information
NR: Non-redundant database
RAxML: Randomized Axelerated Maximum Likelihood
