# Supplementary figures and images for: In silico characterization of a novel putative aerotaxis chemosensory system in the myxobacterium, Corallococcus coralloides
Source: BMC Genomics. 2018 Oct 19;19:757. doi: 10.1186/s12864-018-5151-6 (PMC6194562; doi:10.1186/s12864-018-5151-6)

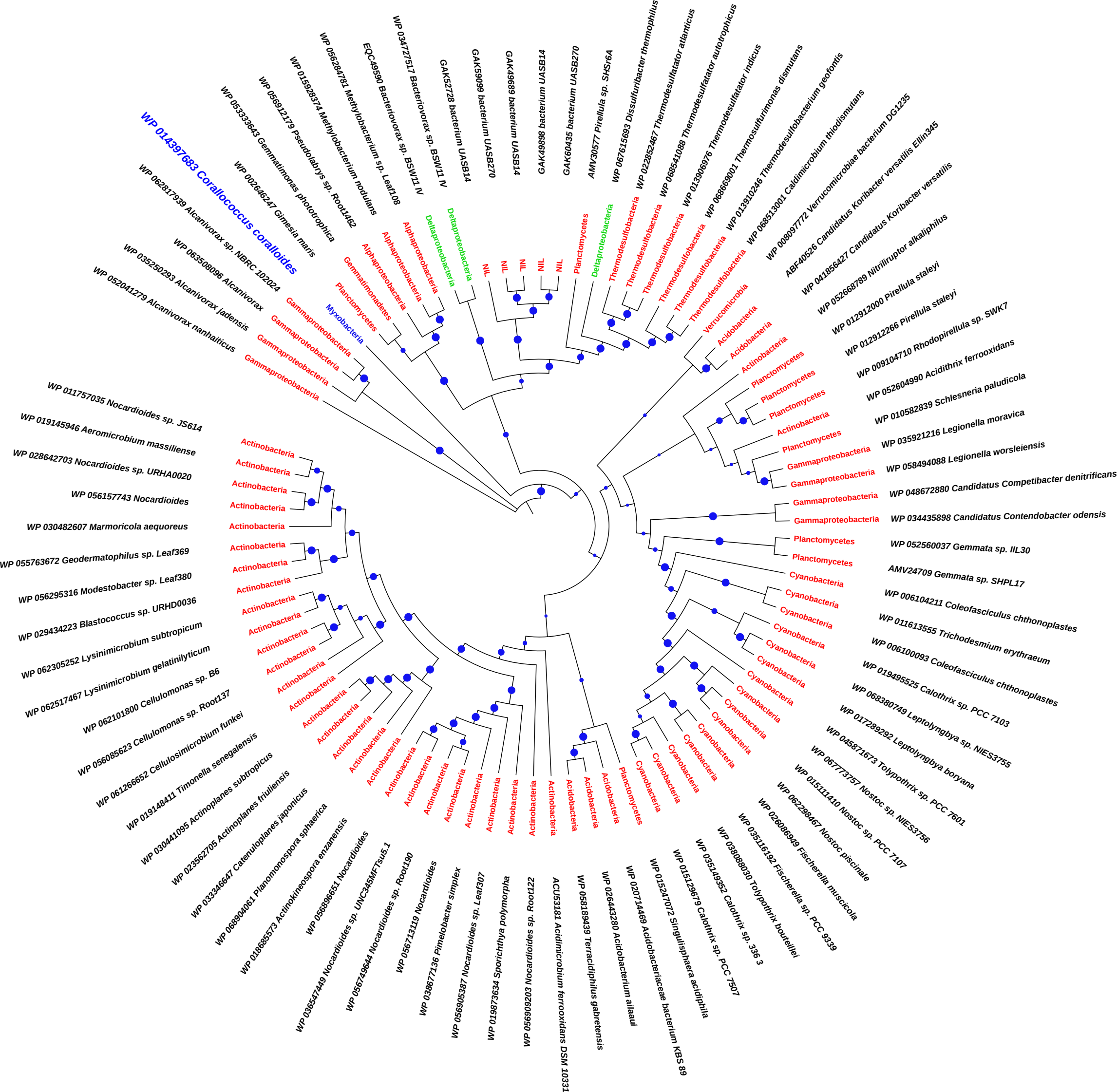

Supplement: Supplementary file 1 — Figure S1. Maximum likelihood phylogeny for CheA protein of the energy taxis cluster in C. coralloides. The top homologs of the CheA protein (Cc_4970) involved in energy taxis in C. coralloides are used here to generate this represented ML phylogenetic tree with organism names in the outermost ring. The taxonomy lineage at the phylum level is represented in the inner ring where myxobacterial homologs are in blue text, non-Myxococcales Deltaproteobacteria in green text and other taxa in red text. Bootstrap values are provided corresponding to the tree nodes as blue circles with sizes ranging from one (BS value 1) to 15 (BS value 100). (PDF 49 kb) [file 12864_2018_5151_MOESM1_ESM.pdf]

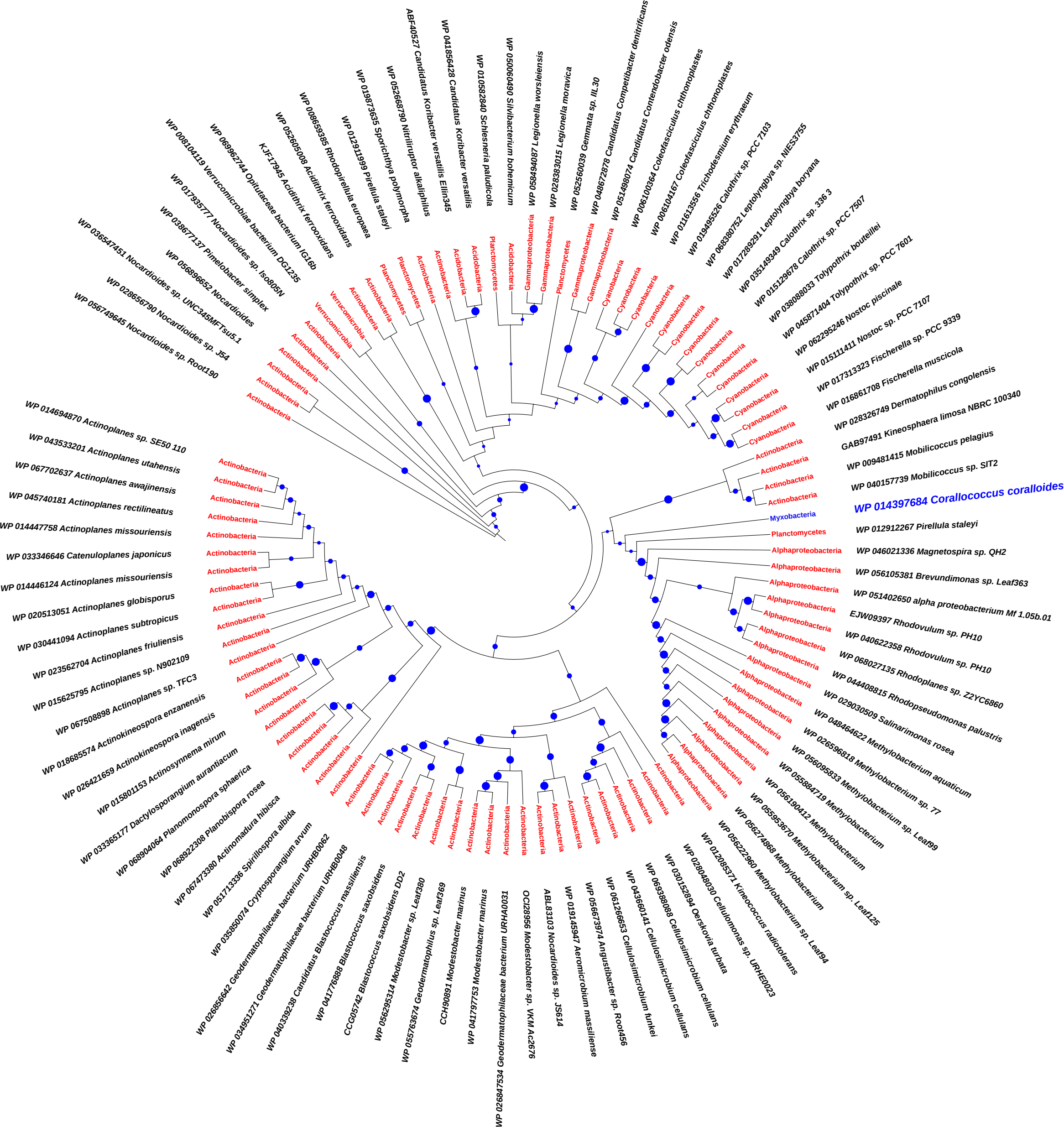

Supplement: Supplementary file 2 — Figure S2. Maximum likelihood phylogeny for CheW protein, a part of the energy taxis cluster in C. coralloides. The top homologs of the CheW protein (Cc_4971) involved in energy taxis in C. coralloides were used to generate this represented ML phylogenetic tree with organism names in the outermost ring. The taxonomy lineage at the phylum level is represented in the inner ring where myxobacterial homologs are in blue text, non-Myxococcales Deltaproteobacteria in green text and other taxa in red text. Bootstrap values are provided corresponding to the tree nodes as blue circles with sizes ranging from one (BS value 1) to 15 (BS value 100). (PDF 51 kb) [file 12864_2018_5151_MOESM2_ESM.pdf]

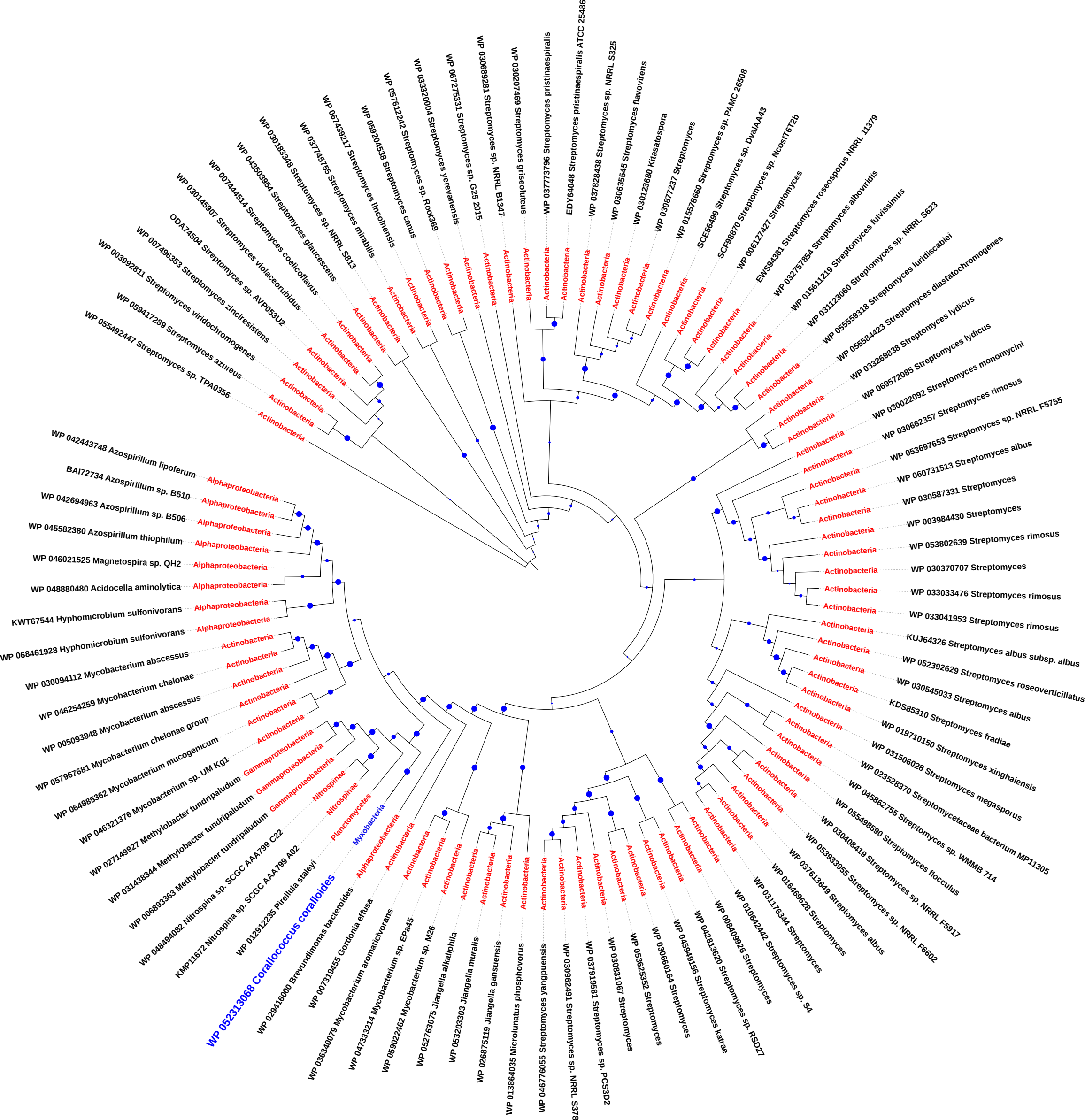

Supplement: Supplementary file 3 — Figure S3. Maximum likelihood phylogeny for the Cc_4973 protein, a constituent of the Cc-5 energy taxis cluster. The top homologs of the Cc_4973 protein, which is encoded in the energy taxis cluster in C. coralloides, were used to generate this represented ML phylogenetic tree with organism names in the outermost ring. The taxonomy lineage at the phylum level is represented in the inner ring where myxobacterial homologs are in blue text, non-Myxococcales Deltaproteobacteria in green text and other taxa in red text. Bootstrap values are provided corresponding to the tree nodes as blue circles with sizes ranging from one (BS value 1) to 15 (BS value 100). (PDF 43 kb) [file 12864_2018_5151_MOESM3_ESM.pdf]

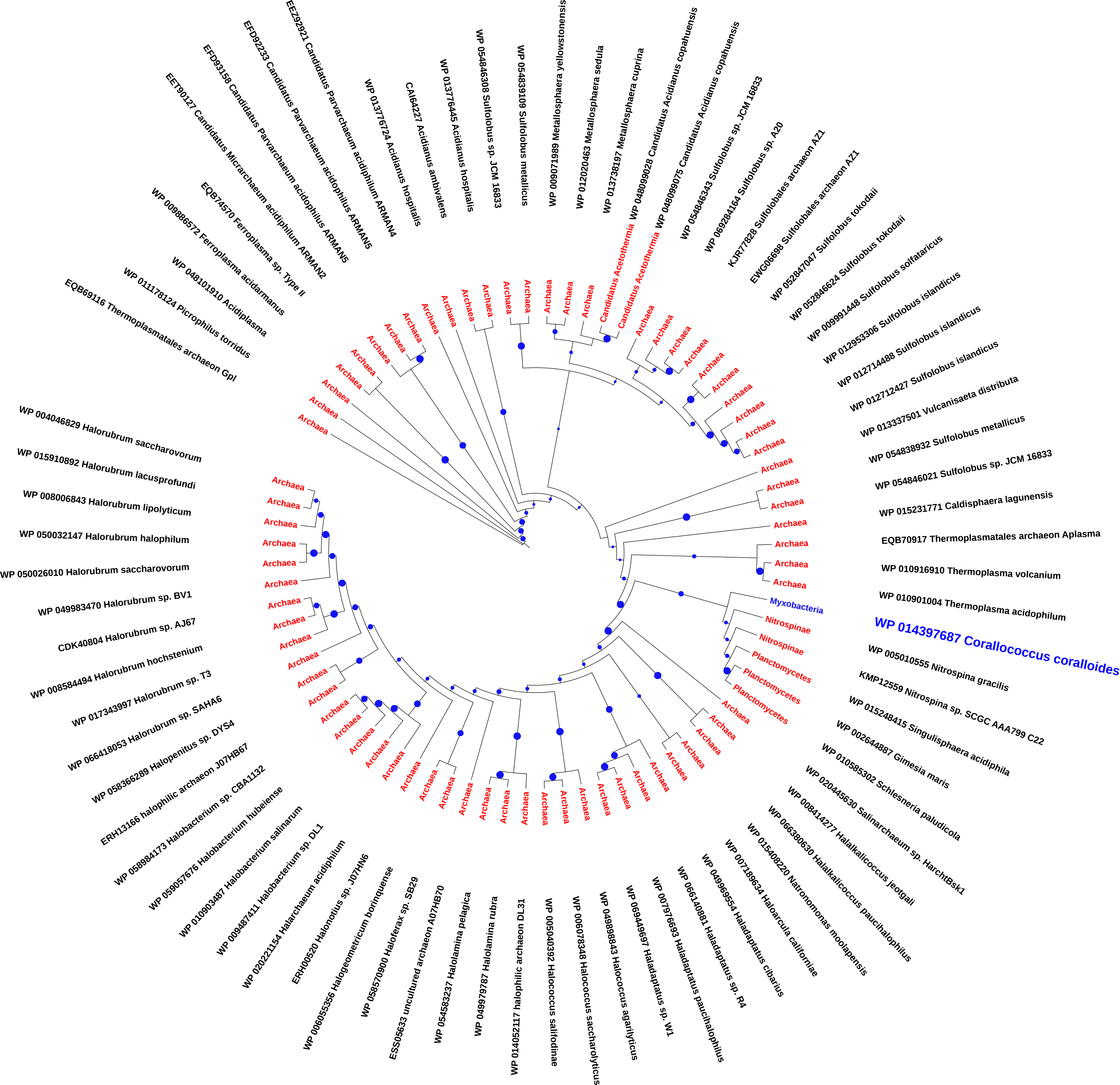

Supplement: Supplementary file 4 — Figure S4. Maximum likelihood phylogeny for the Cc_4974 protein, a constituent of the Cc-5 energy taxis cluster. The top homologs of the Cc_4974 protein, which is encoded in the energy taxis cluster in C. coralloides, were used to generate this represented ML phylogenetic tree with organism names in the outermost ring. The taxonomy lineage at the phylum level is represented in the inner ring where myxobacterial homologs are in blue text, non-Myxococcales Deltaproteobacteria in green text and other taxa in red text. Bootstrap values are provided corresponding to the tree nodes as blue circles with sizes ranging from one (BS value 1) to 15 (BS value 100). (PDF 36 kb) [file 12864_2018_5151_MOESM4_ESM.pdf]

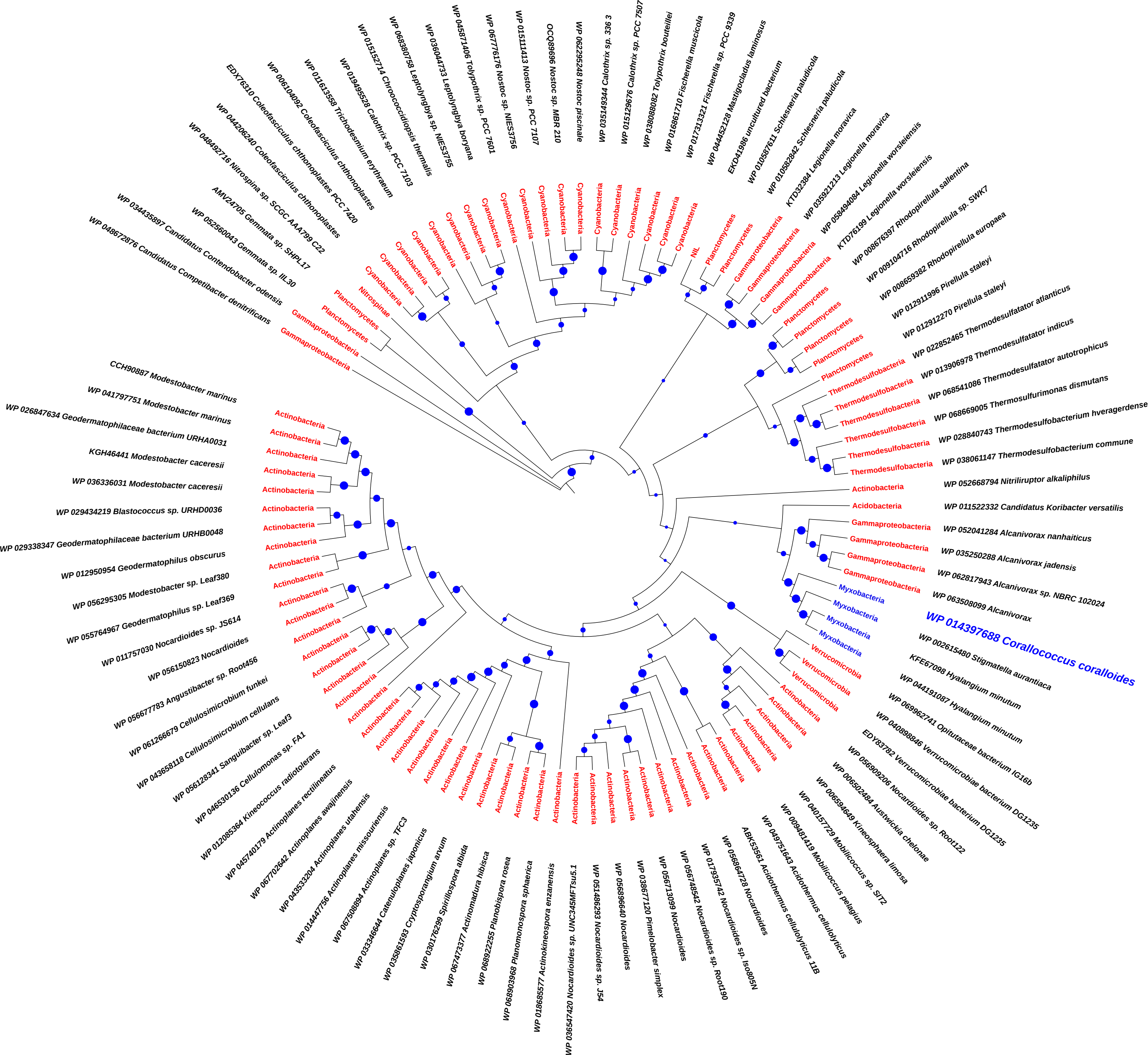

Supplement: Supplementary file 5 — Figure S5. Maximum likelihood phylogeny for the CheB protein involved in the energy taxis cluster in C. coralloides. The top homologs of the CheB protein (Cc_4975) involved in the energy taxis in C. coralloides were used to generate this represented ML phylogenetic tree with organism names in the outermost ring. The taxonomy lineage at the phylum level is represented in the inner ring where myxobacterial homologs are in blue text, non-Myxococcales Deltaproteobacteria in green text and other taxa in red text. Bootstrap values are provided corresponding to the tree nodes as blue circles with sizes ranging from one (BS value 1) to 15 (BS value 100). (PDF 52 kb) [file 12864_2018_5151_MOESM5_ESM.pdf]

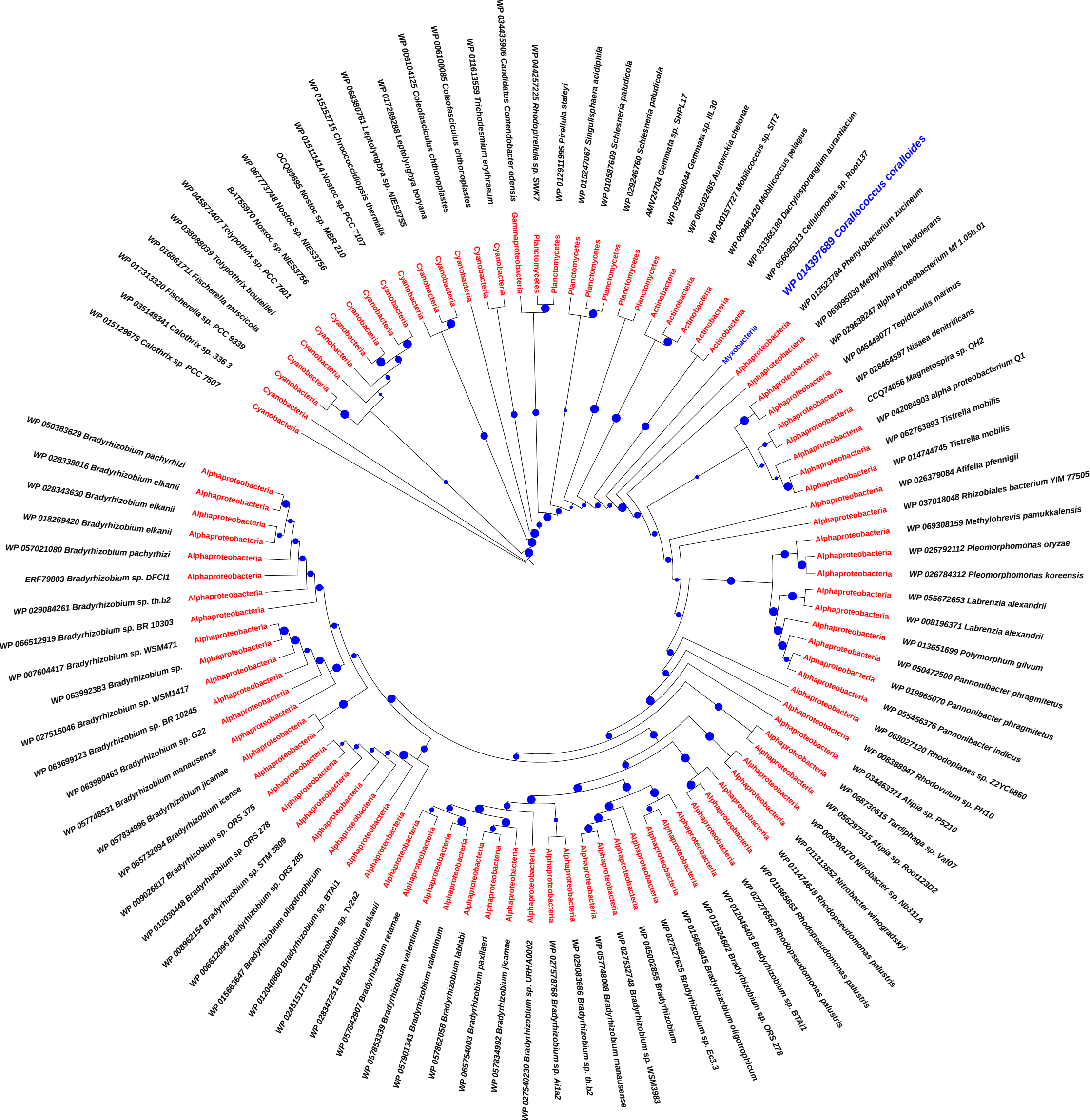

Supplement: Supplementary file 6 — Figure S6. Maximum likelihood phylogenetic tree for the CheR protein in the Cc-5 cluster in C. coralloides. The top homologs of the CheR protein (Cc_4976) involved in energy taxis in C. coralloides were used to generate this represented ML phylogenetic tree with organism names in the outermost ring. The taxonomy lineage at phylum level is represented in the inner ring where myxobacterial homologs are in blue text, non-Myxococcales Deltaproteobacteria in green text and other taxa in red text. Bootstrap values are provided corresponding to the tree nodes as blue circles with sizes ranging from one (BS value 1) to 15 (BS value 100). (PDF 50 kb) [file 12864_2018_5151_MOESM6_ESM.pdf]
